# Cholesterol accumulation in ovarian follicles causes ovulation defects in *Abca1a*^*−/−*^ Japanese medaka (*Oryzias latipes*)

**DOI:** 10.1016/j.heliyon.2023.e13291

**Published:** 2023-01-31

**Authors:** Ryota Futamata, Masato Kinoshita, Katsueki Ogiwara, Noriyuki Kioka, Kazumitsu Ueda

**Affiliations:** a Graduate School of Agriculture, Kyoto University, Kyoto 606-8502, Japan; b Laboratory of Reproductive and Developmental Biology, Faculty of Science, Hokkaido University, Sapporo, Japan; c Institute for Integrated Cell-Material Sciences (WPI-iCeMS), KUIAS, Kyoto University, Kyoto 606-8501, Japan

**Keywords:** Abca1a−/− medaka causes ovulation defects, ABC transporter, ABCA1, Japanese medaka, Cholesterol, Ovary, Ovulation

## Abstract

ATP-binding cassette A1 (ABCA1) is a membrane protein, which exports excess cellular cholesterol to generate HDL to reduce the risk of the onset of cardiovascular diseases (CVD). In addition, ABCA1 exerts pleiotropic effects on such as inflammation, tissue repair, and cell proliferation and migration. In this study, we explored the novel physiological roles of ABCA1 using Japanese medaka (*Oryzias latipes),* a small teleost fish. Three *Abca1* genes were found in the medaka genome. ABCA1A and ABCA1C exported cholesterol to generate nascent HDL as human ABCA1 when expressed in HEK293 cells. To investigate their physiological roles, each *Abca1*-deficient fish was generated using the CRISPR-Cas9 system. *Abca1a*^*−/−*^ female medaka was found to be infertile, while *Abca1b*^*−/−*^ and *Abca1c*^*−/−*^ female medaka were fertile. *In vitro* ovarian follicle culture suggested that *Abca1a* deficiency causes ovulation defects. In the ovary, ABCA1A was expressed in theca cells, an outermost layer of the ovarian follicle. Total cholesterol content of *Abca1a*^*−/−*^ ovary was significantly higher than that of the wild-type, while estrogen and progestin contents were compatible with those of the wild-type. Furthermore, cholesterol loading to the wild-type follicles caused ovulation defects. These results suggest that ABCA1A in theca cells regulates cholesterol content in the ovarian follicles and its deficiency inhibits successful ovulation through cholesterol accumulation in the ovarian follicle.

## 1 Introduction

The ATP-binding cassette (ABC) protein superfamily is one of the largest protein families that mediate the ATP-driven translocation of various substrates across membranes in every living organism [1,2]. In humans, there are 48 ABC protein genes divided into seven subfamilies, and defects in their function are related to various diseases [1,3]. ABCA subfamily members play important roles in cholesterol homeostasis and membrane lipid trafficking [[4], [5], [6]]. ABCA1 is a full-size ABC protein consisting of two transmembrane domains (TMDs) and two nucleotide-binding domains (NBDs) characterized by Walker-A, Walker-B, and ABC signature motifs [7]. ABCA1 was identified as the disease-causing gene for Tangier disease, a rare genetic disorder that exhibits a severe reduction in plasma high-density lipoprotein (HDL) level and a high incidence of premature cardiovascular disease (CVD) [[8], [9], [10]]. A marked reduction in plasma HDL levels was also reported in ABCA1 KO mice [[11], [12], [13], [14], [15]] and WHAM chicken [13,16,17], which carries a missense mutation in *Abca1* gene. *In vitro* studies revealed that ABCA1 transfers excess cellular cholesterol and phosphatidylcholine to lipid-free apolipoprotein A-I (apoA-I) in serum, thereby generating nascent discoidal HDL particles [18]. Thus, ABCA1 is indispensable for HDL generation in various species. Because plasma HDL levels are inversely correlated with the risk of the onset of CVD [19,20], studies on ABCA1 have long been focused on its protective effects against CVD. However, it has been suggested that ABCA1 plays pleiotropic roles in various tissues. For example, ABCA1 is involved in inflammation [21,22], tissue repair [23], and cell proliferation and migration [[24], [25], [26]], suggesting that the physiological roles of ABCA1 are not fully understood. To investigate these roles, several ABCA1 KO mice lines have been generated [27]. Although these mice showed a marked reduction in plasma HDL levels, differences in the extent and severity of the lipid accumulation in various tissues were reported, suggesting that the genetic background of the mice and the diet greatly influence the phenotype. Therefore, analyzing another *Abca1*-deficient animal model may help elucidate the physiological roles of ABCA1.

Medaka (*Oryzias latipes*) is a small, egg-laying freshwater fish that offers advantages for developmental and reproductive studies. It usually spawns daily under laboratory conditions, with a generation time between 6 and 8 weeks, which is comparable to that of mice. The high optical clarity of the embryo and the synchronous extrauterine development make it easy to follow early developmental processes. Furthermore, transgenic techniques and genome editing methods have been established [[28], [29], [30]]. Thus, medaka has been recognized as a potential model vertebrate. In this study, we explored the novel physiological roles of ABCA1 using medaka. Among three medaka Abca1 genes, *Abca1a*-deficient female were found to be infertile. *In vitro* ovarian follicle culture also suggested that *Abca1a* deficiency causes ovulation defects. The total cholesterol content of *Abca1a*^*−/−*^ ovary was significantly higher than that of wild-type and cholesterol loading to wild-type follicles caused ovulation defects. These results suggest that ABCA1A regulates cholesterol content in the ovarian follicle and this regulation is crucial for successful ovulation.

## 2 Results

### 2.1 Characterization of medaka ABCA1 proteins

**Fig. 1:**
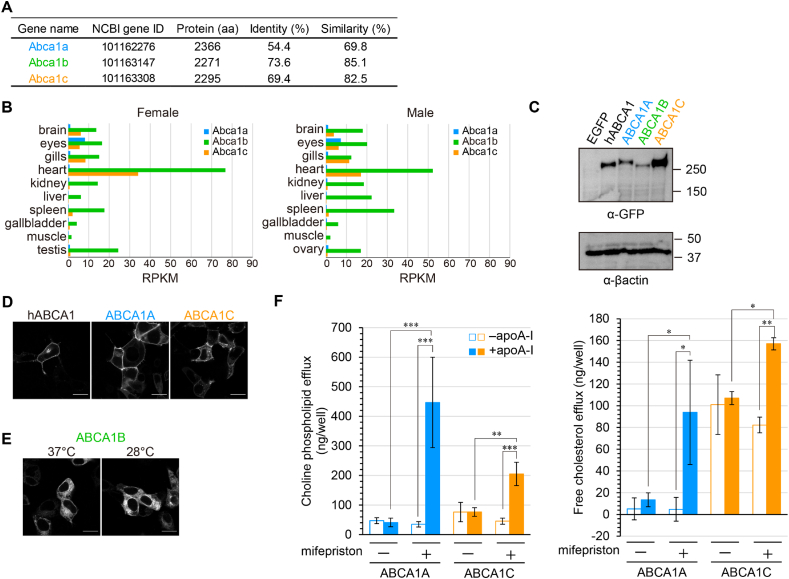
Characterization of medaka ABCA1 proteins (A) List of medaka *Abca1* genes. Amino acid sequences of human ABCA1 (hABCA1) and each medaka ABCA1 protein were compared using EMBOSS Needle. The amino acid sequence of hABCA1 was obtained from the KEGG database. Amino acid sequences of medaka ABCA1 proteins were predicted from cDNA sequences. (B) Tissue distribution of each medaka *Abca1* mRNA. Total RNA was isolated from 8 to 16 fish. RNA-seq data were obtained from a previous study (DDBJ accession number DRA014727) [31]. (C) EGFP-fused hABCA1 or medaka ABCA1 proteins were transiently expressed in HEK293 cells, and EGFP was detected by western blotting. The original blots are shown in figure S2. (D) EGFP-fused proteins were transiently expressed in HEK293 cells, and EGFP fluorescence was detected. Scale bars, 10 μm. (E) Cells were incubated at 37 °C or 28 °C for 3 h before observation. Scale bars, 10 μm. (F) EGFP (control), ABCA1A- EGFP, and ABCA1C- EGFP were transiently expressed in HEK293 cells. The cells were incubated in the absence (open bars) or presence (colored bars) of 10 μg/mL apoA-I at 37 °C for 24 h. Choline phospholipid and free cholesterol released to the medium were quantified by the Amplex Red Enzyme Assay. Experiments were performed in biological triplicate, and mean values are shown ±SD. ***, *p* < 0.001; **, *p* < 0.01; *, *p* < 0.05.

In the medaka genome, three genes were annotated as *Abca1*: *Abca1a*, *Abca1b*, and *Abca1c* (Fig. 1A). The corresponding medaka ABCA1 proteins have high sequence similarity and identity with human ABCA1 (hABCA1) (Fig. 1A). The tissue distribution of each medaka *Abca1* mRNA in wild-type medaka (Cab strain) was investigated by RNA sequencing (RNA-seq) (Fig. 1B). The expression of *Abca1a* was lowest among the three medaka *Abca1* genes but was relatively high in the eyes. *Abca1b* was expressed broadly and had the highest expression. *Abca1c* was highly expressed in the heart, gills, eyes, and brain. To investigate the subcellular localization and function of the medaka ABCA1 proteins, cDNA was prepared from the eyes (for *Abca1a*) and heart (for *Abca1b* and *Abca1c*) and fused with EGFP at the C terminus. Each medaka ABCA1-EGFP protein was expressed in human HEK293 cells. ABCA1A-EGFP and ABCA1C-EGFP were detected at around 250 kDa by western blotting (Fig. 1C) and mainly localized to the plasma membrane (Fig. 1D). Although ABCA1B-EGFP was detected, it was scarcely localized to the plasma membrane and mainly found at the endoplasmic reticulum (Fig. 1E; Left panel). Since medaka lives at temperatures lower than 30 °C, cells were incubated at 28 °C for 3 h. However, the incubation did not change the subcellular localization of ABCA1B-EGFP, with the EGFP fluorescence remaining mainly inside the cells (Fig. 1E; Right panel). When cells expressing hABCA1 are incubated with the lipid acceptor apoA-I, free cholesterol and phosphatidylcholine are released to the medium as nascent HDL particles [18]. Accordingly, free cholesterol and choline phospholipids were released from cells expressing ABCA1A-EGFP or ABCA1C-EGFP to the medium in the presence of 10 μg/mL apoA-I (Fig. 1F), suggesting that ABCA1A and ABCA1C export cholesterol and choline phospholipids to generate nascent HDL particles like hABCA1.

### 2.2 Generation of each *Abca1*-deficient medaka

**Fig. 2:**
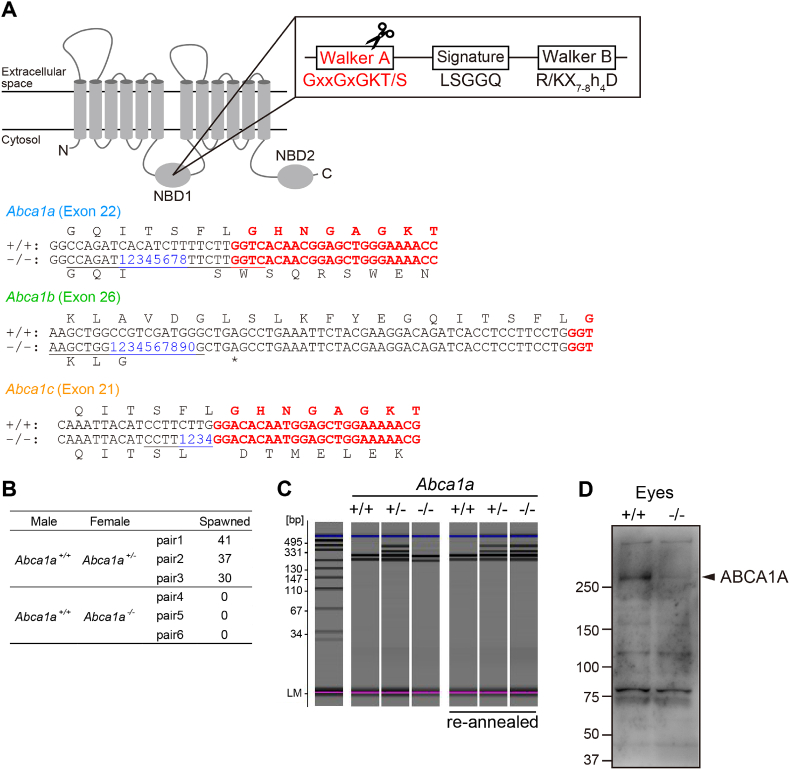
Generation of each *Abca1*-deficient medaka (A) Schematic diagram of the targeted Abca1 gene by the CRISPR-Ca9 system. CrRNA was designed around the Walker A motif in NBD1. The deletion of 8, 10, and 4 nucleotides was confirmed in *Abca1a*, *Abca1b*, and *Abca1c* genes, respectively. (B) *Abca1a* ± and *Abca1a−/−* females were paired with fertile wild-type males, and spawned eggs were counted for three days. Eggs were harvested 3 h after the onset of light. (C) Genotyping PCR. gDNA was extracted from a tail fin, and the *Abca1a* fragment was amplified by PCR. PCR products were analyzed by HMA. The 3 columns on the right represent PCR products, which were reannealed with that of wild-type strain to distinguish *Abca1a*+*/+* and *Abca1a−/−*. (D) *ABCA1A* protein expression in the eyes of *Abca1a*+*/+* and *Abca1a−/−* medaka was analyzed by western blotting. Because the anti-*ABCA1A* antiserum was specific to *ABCA1A*, as described in Materials and Methods, the faint band in *Abca1a−/−* was probably due to nonspecific binding. The original blot and gel are shown in figure S3.

To explore novel physiological roles of ABCA1 in medaka, *Abca1*-deficient medaka were generated using the CRISPR-Cas9 system. The Walker-A motif in the cytosolic nucleotide-binding domain (NBD) is essential for ATP binding [32,33], and mutations in this motif result in the loss of function [34]. Therefore, a CRISPR RNA (crRNA) target sequence was designed around the Walker-A motif in NBD1 (Fig. 2A). Two types of RNA (crRNA, tracrRNA) and Cas9 endonuclease were injected into the cytosol of eggs at the one-cell stage. Then, G_0_ founders that carried *Abca1* mutation in germ cells were mated with wild-type, and F_1_ heterozygotes were obtained. Subsequently, F_1_ heterozygotes were mated with each other, and F_2_ homozygous *Abca1*-deficient medaka were obtained. Each *Abca1* gene mutation was confirmed by DNA sequencing analysis: 8, 10, and 4 nucleotide deletions around the Walker-A motif were found in *Abca1a-*, *Abca1b-*, and *Abca1c*-deficient medaka, respectively (Fig. 2A), and expected to cause premature termination. Next, we investigated whether each *Abca1*-deficient medaka had any phenotypic defects. *Abca1b*^*−/−*^ medaka were born normally and fertile and had a normal appearance. *Abca1c*^*−/−*^ medaka were also born normally, fertile, and had a normal appearance except for fair skin. *Abca1a*^*−/−*^ medaka developed and grew normally (Table 1). However, *Abca1a*^*−/−*^ female medaka were infertile (Fig. 2B). Thus, hereafter, we focused on *Abca1a*^*−/−*^ medaka. The *Abca1a*^*−/−*^ medaka line was maintained by crossing *Abca1a*^+/−^ medaka. The genotype of each fish was confirmed by a heteroduplex mobility assay (Fig. 2C). ABCA1A protein expression disappeared from the eyes of *Abca1a*^*−/−*^ medaka (Fig. 2D).

**Table 1:** Genotypes of *Abca1a* offspring were close to the Mendelian ratio.

	*Abca1a*^*+/−*^ (M) × *Abca1a*^*+/−*^ (F)				*Abca1a*^*−/−*^ (M) × *Abca1a*^*+/−*^ (F)			
	6dpf		adult		6dpf		adult	
	number	(%)	number	(%)	number	(%)	number	(%)
*Abca1a*^*+/+*^	50	24.8	84	25.5	–	–	–	–
*Abca1a*^*+/−*^	96	47.5	167	50.6	60	52.2	124	57.7
*Abca1a*^*−/−*^	56	27.7	79	23.9	55	47.8	91	42.3
Total	202		330		115		215	

### 2.3 *Abca1a* deficiency caused ovulation defects

**Fig. 3:**
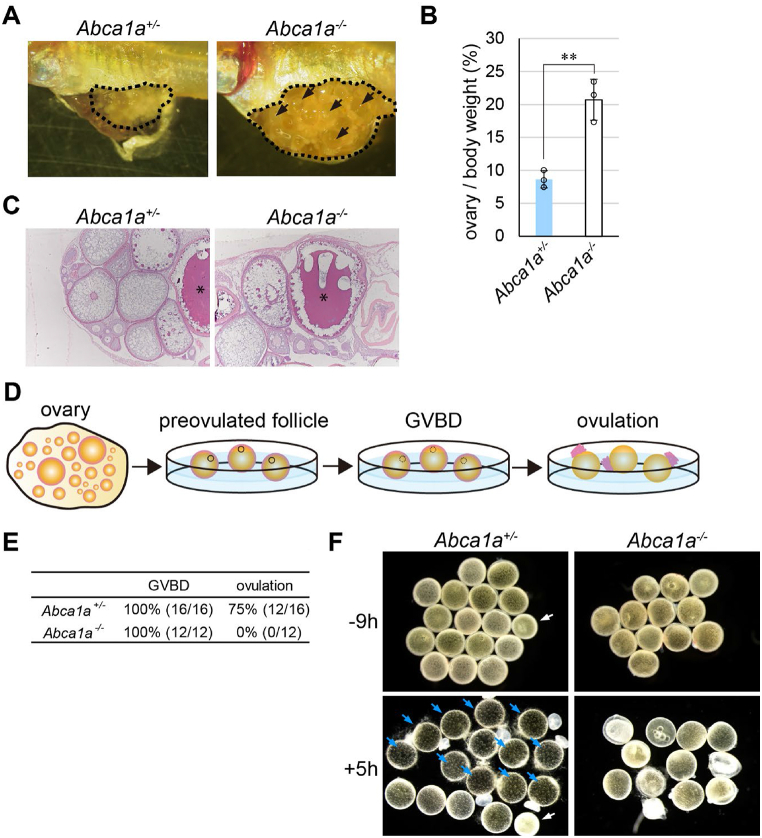
Female infertility in *Abca1a*-deficient medaka (A) *Abca1a* ± and *Abca1a−/−* ovaries. Large follicles that failed to ovulate are indicated by arrows. (B) Ovary weight to bodywght ratio of *Abca1a+/−*; 22–23 wph. *Abca1a−/−*; 18–29 wph. N = 3 fish. Mean values are shown ± SD. (C) Histological analysis of *Abca1a* ± and *Abca1a−/−* ovaries. 10–11 wph. The ovaries were isolated 4 h after the onset of light. *, Normally developed large follicles. (D) Schematic diagram of the *in vitro* follicle culture. (E) Frequencies of GVBD and ovulation. (F) Upper panels, preovulatory follicles isolated from *Abca1a* ± and *Abca1a−/−* ovaries 9–10 h before the onset of light and incubated in 90% Media 199 containing 10 μg/mL gentamycin at 28 °C under 5% CO2. Lower panels, follicles incubated for 5 h after the onset of light. Ovulated eggs were determined from postovulatory follicles and/or many short villi on the surface of the egg membrane. Ovulated eggs are indicated by the blue arrows. The small follicle (<1.0 μm in diameter) indicated by the white arrow was excluded.

The ovary of *Abca1a*^*−/−*^ medaka was found to be enlarged compared to that of *Abca1a*^*+/−*^ medaka (Fig. 3A), and the ovary weight to body weight ratio was more than two-fold higher (Fig. 3B). Since normally developed large follicles in the post-vitellogenic phase (St. IX according to Iwamatsu’s criteria [35]) were observed in *Abca1a*^*−/−*^ ovaries by histological analysis (Fig. 3C), suggesting that oocyte growth proceeded normally. Thus, we hypothesized that *Abca1a* deficiency caused defects in oocyte maturation or ovulation to result in female infertility. To test this hypothesis, oocyte maturation and ovulation were evaluated by an *in vitro* follicle culture. As in mammals, oocyte maturation and ovulation were induced by gonadotropin secreted from the pituitary in teleost fish including medaka. In medaka, follicles destined to ovulate the next morning proceed to final maturation in response to a luteinizing hormone (LH) surge. After this LH surge, follicles destined to ovulate spontaneously proceed to maturation and ovulation *in vitro* [36]. *In vitro* oocyte maturation and ovulation were evaluated by a resumption of meiosis (GVBD; germinal vesicle breakdown) and follicle rupture, respectively (Fig. 3D). Follicles destined to ovulate were isolated from *Abca1a*^*+/−*^ and *Abca1a*^*−/−*^ ovaries 9–10 h before the onset of lighting and incubated at 28 °C. GVBD was examined in all *Abca1a*^*+/−*^ and *Abca1a*^*−/−*^ follicles (Fig. 3E), and 12 out of 16 *Abca1a*^*+/−*^ follicles successfully ovulated (Fig. 3E–F, blue arrows). However, none of the 12 *Abca1a*^*−/−*^ follicles ovulated (Fig. 3E). Furthermore, while *Abca1a*^*−/−*^ follicles seemed to have normal morphology when isolated, some had abnormal morphology after incubation (Fig. 3F). These results suggested that *Abca1a* deficiency caused ovulation defects, resulting in female infertility.

### 2.4 Cholesterol accumulation in *Abca1a*^*−/−*^ follicles caused ovulation defects

**Fig. 4:**
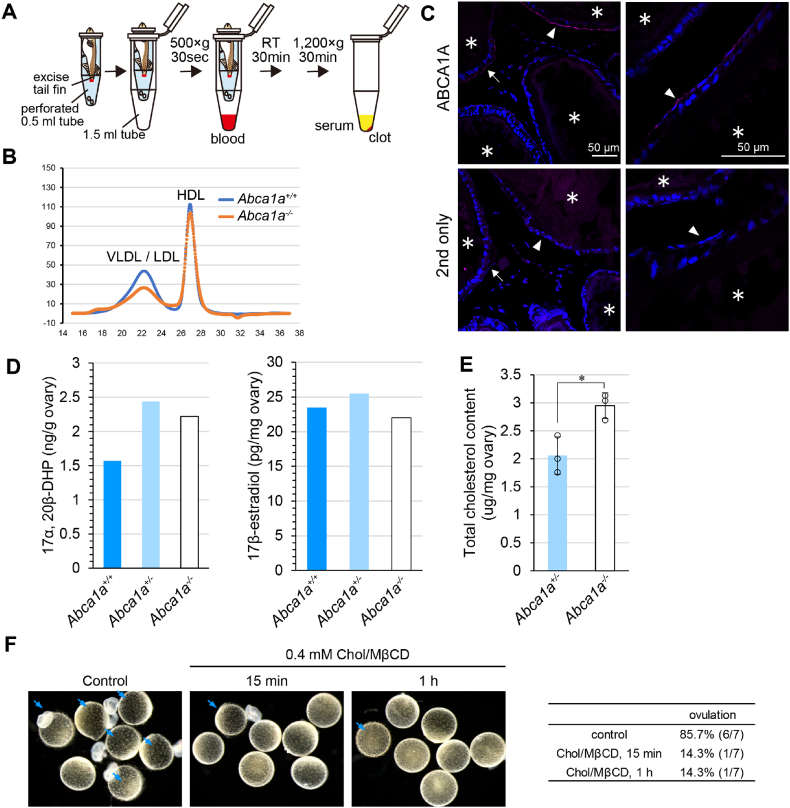
*Abca1a* deficiency disturbed cholesterol homeostasis in the ovary (A) Schematic diagram of the serum collection. See Materials and Methods for details. (B) Serum lipoprotein profiles. Serum from 30 fish at 14–20 wph was pooled. (C) *ABCA1A* protein expression in the ovary was analyzed by immunofluorescent staining. The ovary was isolated 1.5 h after the onset of light. Magenta, *ABCA1A*; Blue, nucleus. Follicles in the late vitellogenic phase and post-vitellogenic phase are indicated by the white arrows and arrowheads, respectively. *, oocyte. Scale bars, 50 μm. (D) Total cholesterol content in *Abca1a* ± and *Abca1a−/−* ovaries. The ovaries were isolated from wild-type fish 3 h after the onset of light. 7–9 wph. N = 3 ovaries. Mean values are shown ±SD. *, p < 0.05. (E) Steroid hormone content in the ovary. The ovaries were isolated 9–9.5 h before the onset of light. The ovaries from 9 fish at 10–17 wph were pooled. (F) Preovulatory follicles were isolated from wild-type ovaries 8.5–10 h before the onset of light and incubated in 90% Media 199 containing 10 μg/mL gentamycin at 28 °C under 5% CO2. Follicles were incubated until 5 h after the onset of light. Ovulated eggs were determined from postovulatory follicles and/or a lot of short villi on the surface of the egg membrane. Ovulated eggs are indicated by the blue arrows.

Next, we investigated how *Abca1a* deficiency caused defects in ovulation. Because ABCA1A exported cholesterol and choline phospholipids in the presence of apoA-I as hABCA1, we speculated that the loss of circulating HDL caused the ovulation defects in *Abca1a*^*−/−*^ medaka. Blood was collected as shown in Fig. 4A, and serum from 30 fish was pooled to analyze the lipoprotein profiles. Unexpectedly, normal HDL generation was observed in *Abca1a*^*−/−*^ medaka (Fig. 4B), suggesting that ABCA1A is not the major contributor to circulating HDL or that ABCA1B or ABCA1C can compensate for blood HDL generation. ABCA1A was detected in theca cells, the outermost layer of follicular cells in large follicles in late-vitellogenic and post-vitellogenic phases according to immunofluorescent staining (Fig. 4C). These results suggested that the ovary-specific function of ABCA1A contributes to successful ovulation rather than to blood HDL production.

In follicular cells, especially in granulosa cells, cholesterol is converted into steroid hormones such as 17β-estradiol and 17α, 20β-dihydroxyprogesterone (17α, 20β-DHP) [37]. These hormones are essential for oocyte growth, oocyte maturation, and ovulation. However, neither the 17β-estradiol nor 17α, 20β-DHP level was decreased in *Abca1a*^*−/−*^ ovaries (Fig. 4D), suggesting that defects in steroid hormone synthesis were not the cause of the ovulation defect in *Abca1a*^*−/−*^ follicles. Instead, interestingly, the total cholesterol content in *Abca1a*^*−/−*^ ovaries was significantly higher than in *Abca1a*^*+/−*^ ovaries (Fig. 4E). We hypothesized that the abnormal cholesterol accumulation in follicles due to *Abca1a* KO caused the ovulation defects. To examine this hypothesis, follicles isolated from wild-type medaka were incubated with 0.4 mM cholesterol methyl beta-cyclodextrin complex (Chol/MβCD) to load cholesterol, and the ovulation rate was assessed (Fig. 4F). While six out of seven control follicles were ovulated, only one out of seven cholesterol-loaded follicles were. These results suggested that cholesterol accumulation in *Abca1a*^*−/−*^ follicles caused the ovulation defects.

## 3 Discussion

Because human ABCA1 is responsible for HDL generation [18] and the plasma HDL level is inversely correlated with the risk of the onset of CVD [19,20], studies on ABCA1 have long been focused on its protective effects against CVD. However, ABCA1 has been suggested to exert pleiotropic effects such as inflammation, tissue repair, and cell proliferation and migration [21,[23], [24], [25], [26],^38^]. In this study, we explored novel physiological roles of ABCA1 using a small teleost fish, Japanese medaka (*Oryzias latipes*).

Medaka has three genes that were annotated as *Abca1*. Whole-genome duplication (WGD) events occurred in the common ancestor of all extant teleost fish [39]. After these WGD events, duplicated genes followed different fates, with the most likely outcome being the loss of one of the duplicates through non-functionalization [39]. In other cases, duplicated genes remained in two copies that divide tissue-specific roles or acquired novel functions. We first investigated the subcellular localization and function of the three medaka ABCA1 proteins using HEK293 cells. ABCA1A and ABCA1C mainly localized to the plasma membrane of HEK293 cells and exported cholesterol and phosphatidylcholine in the presence of apoA-I to the medium, suggesting that they have the same activity as hABCA1 to generate nascent HDL particles, while ABCA1B was scarcely localized to the plasma membrane.

Next, their physiological roles were investigated by generating *Abca1*-deficient medaka using the CRISPR-Cas9 system. *Abca1a*^*−/−*^ female medaka were found to be infertile. Histological analysis and *in vitro* follicle culture suggested that oocyte growth proceeded normally, but defects in ovulation caused female infertility in *Abca1a*^*−/−*^ medaka. Large follicles that failed to ovulate stayed in the ovary (Fig. 3A, allows). *Abca1b*^*−/−*^ medaka were born and grew normally and were fertile. *Abca1c*^*−/−*^ medaka were born normally and fertile but had fair skin. It was reported that WHAM chicken, which carries a missense mutation in *Abca1* gene [17], has white skin and white beaks due to the deficiency in the HDL-mediated transport of carotenoids to the skin [40]. Thus, ABCA1C could be responsible for HDL generation in medaka and *Abca1b* could be a degenerated gene.

In *Abca1*^*−/−*^ mice, severe placental malformation, intrauterine growth retardation in embryos [12], and perinatal lethality of pups [11] have been observed, suggesting that ABCA1 plays an important role in reproduction. However, the relationship between ABCA1 and ovulation has not been reported. Therefore, we focused on *Abca1a*^*−/−*^ medaka to explore the physiological role of ABCA1. In steroidogenic tissues, such as the ovary, cholesterol is converted into steroid hormones. In medaka, granulosa cells, the innermost layer of follicular cells, produce 17α, 20β-dihydroxyprogesterone (17α, 20β-DHP), which is the naturally occurring maturation-inducing hormone, around 14 h before ovulation [41,42] in response to LH secreted from the pituitary. Because 17α, 20β-DHP is essential for ovulation [37], we thought the functional defect of ABCA1A affected the production of this hormone. However, neither 17α, 20β-DHP nor 17β-estradiol content was changed in *Abca1a*^*−/−*^ ovaries (Fig. 4D).

**Fig. 5:**
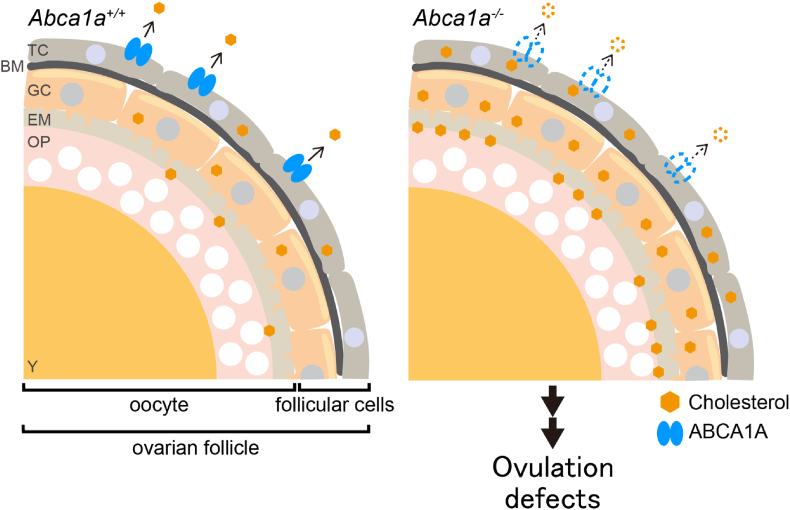
Schematic representation of ovulation defects in *Abca1a*^*−/−*^ medaka ABCA1A regulates cholesterol content in the follicles. Loss of ABCA1A in theca cells causes cholesterol accumulation in the follicles to inhibit successful ovulation. TC, theca cells; BM, basement membrane; GC, granulosa cells; EM, egg membrane; OP, ooplasm; Y, yolk.

It has been suggested that a disturbance of cholesterol homeostasis causes female infertility. For example, mice deficient in SR-BI, a cell surface HDL receptor, show female infertility [43]. In SR-BI KO mice, a high (approximately two-fold) level of plasma total cholesterol, most of which was constituted of abnormally large, heterogeneous, apoE-enriched HDL-like particles, was observed [43]. In addition, denuded SR-BI KO eggs have an abnormally high level of cholesterol, and cholesterol loading to denuded wild-type eggs causes premature egg activation [44], suggesting that excess cholesterol in eggs might cause infertility in SR-BI KO female mice. In *Abca1a*^*−/−*^ medaka, circulating HDL was normal (Fig. 4B). However, interestingly, the cholesterol content in *Abca1a*^*−/−*^ ovary was significantly higher than that in *Abca1a*^*+/−*^ ovary (Fig. 4E). Furthermore, cholesterol loading to wild-type preovulatory follicles inhibited ovulation (Fig. 4F). Immunostaining analysis suggested that ABCA1A was expressed in theca cells, the outermost layer of follicular cells, in preovulatory large follicles (Fig. 4C). These results suggested that the loss of ABCA1A in theca cells prevented the export of cholesterol and caused cholesterol accumulation in the follicles to inhibit successful ovulation (Fig. 5). In mice, denuded ovulated oocytes from *Abca1*-deficient mice had excess cholesterol and lower viability than wild-type oocytes, suggesting that ABCA1 regulates cholesterol homeostasis in oocytes and maintains the quality of oocytes in mammals [45].

In this study, we explored the physiological role of ABCA1 using Japanese medaka. In medaka, at least two of three ABCA1 proteins, ABCA1A and ABCA1C, were found to be functional. Because the circulating HDL level was normal in *Abca1a*^*−/−*^ medaka, we concluded ABCA1A is not a major contributor to circulating HDL. However, additional study found it does play an important role in the follicles to regulate cholesterol content for successful ovulation. The role of cholesterol and other sterols in male and female reproduction has been intensively studied [43,44,[46], [47], [48], [49], [50]], but more detail is needed. This study sheds light on a novel role of ABCA1 in female reproduction.

*Abca1a*^*+/−*^ male and *Abca1a*^*+/−*^ female were crossed, and genotypes of their offspring were analyzed. Similarly, *Abca1a*^*−/−*^ males and *Abca1a*^*+/−*^ females were crossed, and genotypes of their offspring were analyzed. Dpf, days after fertilization.

## 4 Materials and Methods

### 4.1 cDNA cloning and plasmid construction

The cDNA encoding the open reading frame of medaka *Abca1a* (Gene ID: 101162276) gene was amplified by PCR using medaka eye cDNA as the template. *Abca1b* (Gene ID: 101163147) and *Abca1c* (Gene ID: 101163308) genes were amplified by PCR using medaka heart cDNA as the template. Each cDNA was inserted into pEGFP-C1 so that EGFP was fused with the C-terminus of each *Abca1* gene. For expression in BHK/pSwitch cells [51], EGFP-fused *Abca1a* and *Abca1c* cDNA were inserted into pGene/V5-His A (blasticidin) [52]. The sequences of all plasmids were confirmed by Sanger sequencing.

### 4.2 Cell culture

HEK293 cells (ACTT® CRL-1573™) were cultured in Dulbecco’s Modified Eagle Medium (DMEM; Nacalai Tesque) supplemented with 10% heat-inactivated fetal bovine serum (FBS; Gibco) at 37 °C in a humidified atmosphere containing 5% CO_2_. BHK/pSwitch cells [51] were cultured in DMEM supplemented with 10% heat-inactivated FBS and 350 μg/mL hygromycin B (FUJIFILM Wako) at 37 °C in a humidified atmosphere containing 5% CO_2_.

### 4.3 Western blotting

HEK293 cells were seeded in 6-well plates coated with Poly-l-lysine (PLL; Sigma-Aldrich) at 3.0 × 10^5^ cells/well and incubated for 24 h. The cells were transfected with each expression vector using Lipofectamine LTX with PLUS reagent (Invitrogen) according to the manufacturer’s instructions and then further incubated for 6 h. Then, the medium was replaced with DMEM supplemented with 10% FBS and incubated for another 18 h. The cells were lysed with 1% Triton X-100 containing 1% cOmplete™ Protease Inhibitor Cocktail (Roche). Tissues were homogenized in lysis buffer (20 mM Tris-HCl pH7.4 at 4 °C, 1% Triton X-100, 0.1% SDS, 1% Sodium deoxycholate, 150 mM NaCl) containing 1% protease inhibitor cocktail. Samples were electrophoresed on SDS-polyacrylamide gels, transferred to polyvinylidene difluoride membrane, and probed with the indicated primary antibodies. Secondary antibodies conjugated to horseradish peroxidase were detected using an Immunostar LD/Zeta (FUJIFILM Wako). Anti-GFP monoclonal antibody (B-2, catalog number: sc-9996) was purchased from Santa Cruz Biotechnology. Anti-ABCA1A antiserum was generated against 19 amino acids (EPGKKRRRKKEPLETDLLS) in the ABCA1A linker region between NBD1 and TMD2. The amino acid sequence of the epitope is quite different from ABCA1B and ABCA1C, as shown in Figure S1. The antibody was made at Sigma-Aldrich Japan.

### 4.4 Microscopy

HEK293 cells were seeded in a 35-mm glass base dish (IWAKI) coated with PLL at 3.0 × 10^5^ cells/dish and incubated for 24 h. The cells were transfected with each expression vector using PEI-MAX (MW = 40,000, Polysciences), as described previously [53], and further incubated for 23 h. The medium was replaced with FluoroBrite DMEM (Gibco) supplemented with 10% FBS, 1 mM sodium pyruvate (Gibco), and 1 × GlutaMAX (Gibco) and incubated for another 1 h. Fluorescent images were obtained at 37 °C under 5% CO_2_ using a confocal laser scanning microscope (Carl Zeiss, model: LSM700) operated by Zen 2012 and equipped with a Plan-Apochromato × 63/1.4 NA oil immersion objective lens, Incubator PM S1, Heating Insert P S1, TempModule S1, and CO_2_ Module S1.

### 4.5 Cholesterol and phospholipids efflux assay

BHK/pSwitch cells were seeded in 6-well plates coated with PLL at 4.0 × 10^5^ cells/well and incubated for 24 h. The cells were transfected with each expression vector (pGENE/V5-His) using Lipofectamine LTX with PLUS reagent and incubated for 24 h. Protein expression was induced by incubation with 10 nM mifepriston (GeneSwitch system, Invitrogen) for another 24 h. Then, the medium was replaced with DMEM containing 0.02% BSA and 10 μg/mL apoA-I, and the cells were incubated for another 24 h. The lipids were extracted from the cultured medium with chloroform/methanol (2:1). The lipid solution was dried and resuspended in reaction mixture (0.01% Triton X-100, 5 mM sodium cholate). For cholesterol measurements, 50 μL of the lipid solution was transferred to a black 96-well plate, 50 μL of Hank’s balanced salt solution (HBSS; Gibco) containing 20 mU cholesterol oxidase, 2 mU HRP, and 50 pmol Amplex Red was added, and the mixture was incubated at 37 °C. For choline phospholipid measurements, 40 μL of the lipid solution was transferred to a black 96-well plate, 10 μL of reaction mixture containing 5 U phospholipase D and 50 μL HBSS containing 20 mU choline oxidase, 2 mU HRP, and 50 pmol Amplex Red was added, and the mixture was incubated at 37 °C. Subsequently, the fluorescence intensity (Ex. 535 ± 20 nm/Em. 590 ± 20 nm) was measured using a microplate reader (Biotek, model: Cytation 5).

### 4.6 Medaka

This study was performed in compliance with the Regulation for Animal Experiments in Kyoto University. All efforts were made to minimize suffering. The Cab inbred closed colony was used in this study. Medaka were maintained in an aquarium with recirculating water in a 14/10 h light/dark cycle at 26 °C. Medaka was fed on artemia once a day and OTOHIME (Marubeni Nisshin Feed) once or twice a day.

### 4.7 Genotyping

The genotypes were confirmed by PCR using genomic DNA (gDNA). gDNA was extracted, as described previously [31]. The DNA fragments containing each target site were amplified by PCR using KOD-FX and specific primers (*Abca1a*: 5’-TACTAGATAACATTGAGGCAG-3’ and 5’-GCTGCGGAGTATAAGTCGCAC-3’; *Abca1b*: 5’-TGTGCATTGAGGAGGAACCCG-3’ and 5’-GTCCTTCCCGAGGATGTAAGC-3’; and *Abca1c*: 5’-GTTTGTGTGGAGGAGGAGCCTG-3’ and 5’-TCCCAAGATGTAGGCGGTGCCAG-3’) or BIOTAQ and specific primers (*Dmy*: 5’-CCGGGTGCCCAAGTGCTCCCGCTG-3’ and 5’-GATCGTCCCTCCACAGAGAAGAGA-3’). The resulting amplicons were analyzed using a microchip electrophoresis system (Shimadzu, model: MCE-202 MultiNA) with a DNA-500 reagent kit for *Abca1a*, *Abca1b*, and *Abca1c* or 1% agarose electrophoresis for *Dmy*.

### 4.8 Microinjection

The injection mixture (50 ng/μL of crRNA, 80 ng/μL of tracrRNA, and 500 ng/μL of Cas9 protein) was prepared. Approximately 2–4 nL of each injection mixture was injected into the cytosol of eggs at the one-cell stage, as described previously [30]. The crRNA and tracrRNA sequences were as follows. *Abca1a*: GACCAAGAAAAGAUGUGAUCGUUUUAGAGCUAUGCUGUUUUG; *Abca1b*: GAAACAAGCUGGCCGUCGAUGUUUUAGAGCUAUGCUGUUUUG; *Abca1c*: CCGUCAAGGUAAGAAGCUAGGUUUUAGAGCUAUGCUGUUUUG; and tracrRNA: AAACAGCAUAGCAAGUUAAAAUAAGGCUAGUCCGUUAUCAACUUGAAAAAGUGGCACCGAGUCGGUGCU.

### 4.9 Serum lipoprotein profile analysis

Blood samples were obtained from medaka following a fast more than 12 h long. Fish were kept on ice for 1 min and then bled by cutting a ventral portion around the tail fin. A fish was put with the wound side down into a 0.5 mL tube, which was previously perforated with a needle. The edge of the holes in the tube was smoothened after a brief exposure to a lighter frame. Then, a 0.5 mL tube containing the amputated fish was placed into a 1.5 mL tube and centrifuged at 500×*g*, 4 °C for 30 s. Blood samples from 30 fish were pooled and kept at room temperature for 30 min. Following this, blood samples were further centrifugated at 1,200×*g*, 4 °C for 30 min to separate serum and clot. The serum samples were immediately frozen with liquid nitrogen and stored at −80 °C until analysis. Cholesterol and TG profiles were analyzed by a gel-permeation high-performance liquid chromatography (GP-HPLC) system (LipoSEARCH®), as described previously [54]. Analysis was performed at Immuno-Biological Laboratories.

### 4.10 Steroid hormone analysis

Ovaries were isolated 8.5–9 h before the onset of light and immediately frozen with liquid nitrogen. Collected samples were stored at −80 °C until analysis. Ovarian 17β-estradiol and 17α,20β-DHP content was measured by LC-MS/MS. Lipids were extracted using *tert*-butyl methyl ether and purified using Oasis MAX (Waters). Lipids were derivatized to picolinyl esters by a reaction with picolinic acid for the detection of poorly ionized steroids, especially those of low abundance. Analytes were separated by the Nexera UPHPLC system (Shimadzu) and detected with an API 4000 triple quadrupole system (Sciex). Positive electrospray ionization mode was used. Lipid extraction and analysis by LC-MS/MS were performed at Aska Pharma Medical.

### 4.11 *In vitro* follicle culture

Ovaries were isolated 9–10 h before the onset of light, and large follicles (>1.0 mm in diameter) were isolated in HBSS. Then, the follicles were transferred to 90% Media 199 containing 10 μg/mL gentamycin (Gibco) and incubated at 28 °C in a humidified atmosphere containing 5% CO_2_. GVBD and follicle rupture were evaluated using an ordinary light microscope.

### 4.12 Histochemistry

Ovaries were isolated 4 h after the onset of light and fixed in Bouin’s fixative at 4 °C. After dehydration, 4-μm thick plastic sections were prepared using a Technovit 8100 (Heraeus Kulzer, catalog number: RT8100) following the manufacturer’s instructions. The plastic sections were stained with hematoxylin-eosin.

### 4.13 Immunofluorescence staining

Ovaries were isolated 1.5 h after the onset of light and fixed in 4% paraformaldehyde at 4 °C. After dehydration, samples were embedded in paraffin and sectioned into 5-μm thick slices. After deparaffinization, tissue sections were incubated in 10 μg/mL Proteinase K at 37 °C for 10 min for antigen retrieval and 5% Blocking One Histo (Nacalai tesque) at room temperature for 5 min to reduce nonspecific antibody binding. Then, tissue sections were probed with anti-ABCA1A polyclonal antibody and subsequently probed with Anti-Rabbit IgG antibody conjugated to Alexa Fluor® 555 (Thermo). Fluorescent images were obtained using a Nikon C2 confocal system.

### 4.14 Statistical analysis

Statistical analyses were performed using Welch’s *t-test* or Tukey’s post hoc test following one-way ANOVA. All statistical analyses were performed using Origin2018 software (Lightstone).

## Author contribution statement

Ryota Futamata: Conceived and designed the experiments; Performed the experiments; Analyzed and interpreted the data; Wrote the paper.

Masato Kinoshita: Conceived and designed the experiments; Performed the experiments; Analyzed and interpreted the data; Contributed reagents, materials, analysis tools or data.

Katsueki Ogiwara; Noriyuki Kioka: Analyzed and interpreted the data.

Kazumitsu Ueda: Conceived and designed the experiments; Analyzed and interpreted the data; Wrote the paper.

## Funding statement

Profs Kazumitsu Ueda and Masato Kinoshita were supported by 10.13039/501100001691Japan Society for the Promotion of Science [18H05269].

## Data availability statement

Data will be made available on request.

## Declaration of interest's statement

The authors declare no conflict of interest.

## Animal experiments

These experiments were approval by the animal ethics committee of Kyoto University (R4-45) and conducted according to established animal welfare guidelines.
