# Supplementary material for: Cholesterol accumulation in ovarian follicles causes ovulation defects in Abca1a−/− Japanese medaka (Oryzias latipes)
Source: Heliyon. 2023 Jan 31;9(2):e13291. doi: 10.1016/j.heliyon.2023.e13291 (PMC9932449; doi:10.1016/j.heliyon.2023.e13291)
Supplement: Multimedia component 1 [file mmc1.docx]

Cholesterol accumulation in ovarian follicles causes ovulation defects in Abca1a-/- Japanese medaka (Oryzias latipes)

Ryota Futamata, Masato Kinoshita, Katsueki Ogiwara, Noriyuki Kioka, and Kazumitsu Ueda*

Supplemental Figures S1-S3

ABCA1A 1223 GSLAIFLSKLDQRLPELGISSYGLSDSTLEEIFLRVAEETGVDAEPEQEELSPNSQQPSG 1282

ABCA1B 1224 GAFVELFHELDDRLTDLGISSYGISDTTLEEIFLKVAEDSGVDAVELSDGVVPTRTRRRH 1283

ABCA1C 1239 GAFVELFKDLDLKLHELGISSYGVSDTTLEEIFLKVAEDNGVDTEVLSDGTLPVRARRRT 1298

*::. :: .** :* :*******:**:*******:***:.***: .: * :

ABCA1A 1283 ERV---EEQPAEQPEP------GKKRRRKKEPLETDLLSGDCRGGE-PLSGSWLMWQQLK 1332

ABCA1B 1284 -A--FGDHQSCLKPFTED-EFDFNDSEGDPESRETDWLGGADGKGSFQVKGWSLKRQQFV 1339

ABCA1C 1299 HAFGGGEHQSCLKPISEDDTYDCNDSEGDPDCRETDWLSCTEGKGSYQVNGWSLRRQQFV 1358

:.* . :* :. . . : *** *. *. :.* * **:

Figure S1. Multiple alignments of amino acid sequences among medaka ABCA1 proteins.

Amino acid sequences around the linker region between NBD1 and TMD2 are shown. The epitope against anti-ABCA1A antiserum and polyclonal antibody is surrounded by the red circle.


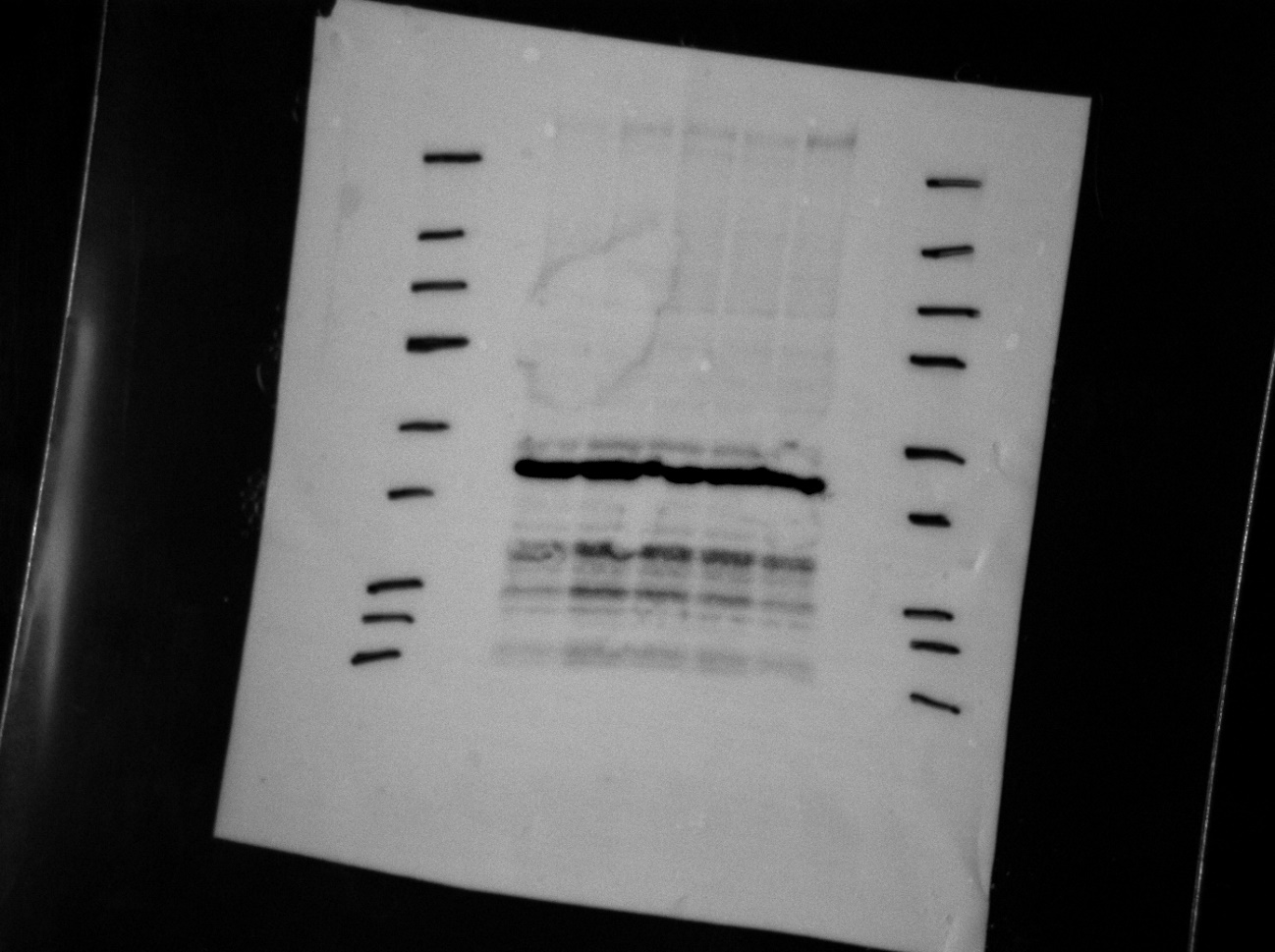

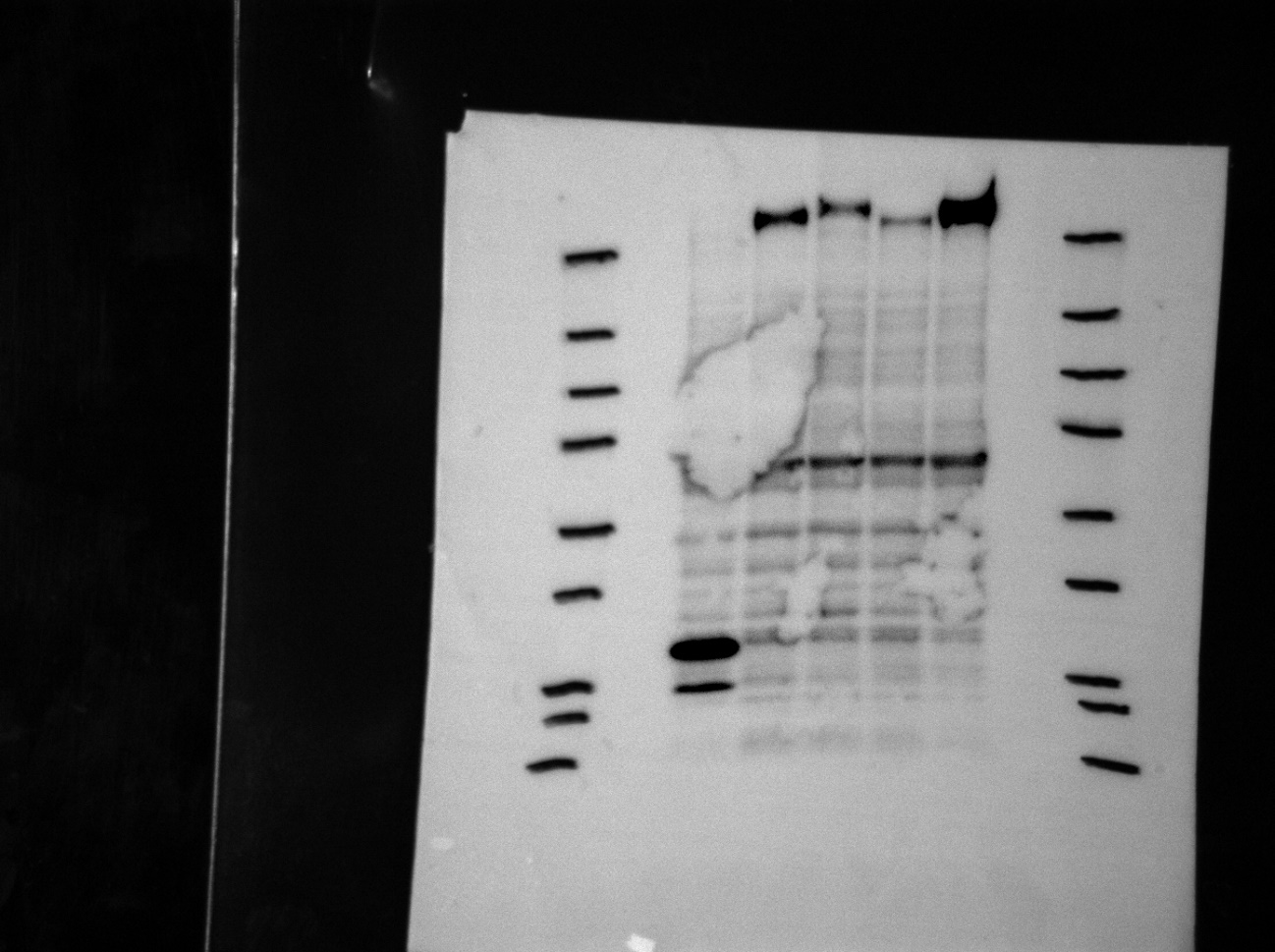
Figure S2. The original blots of Figure 1C.





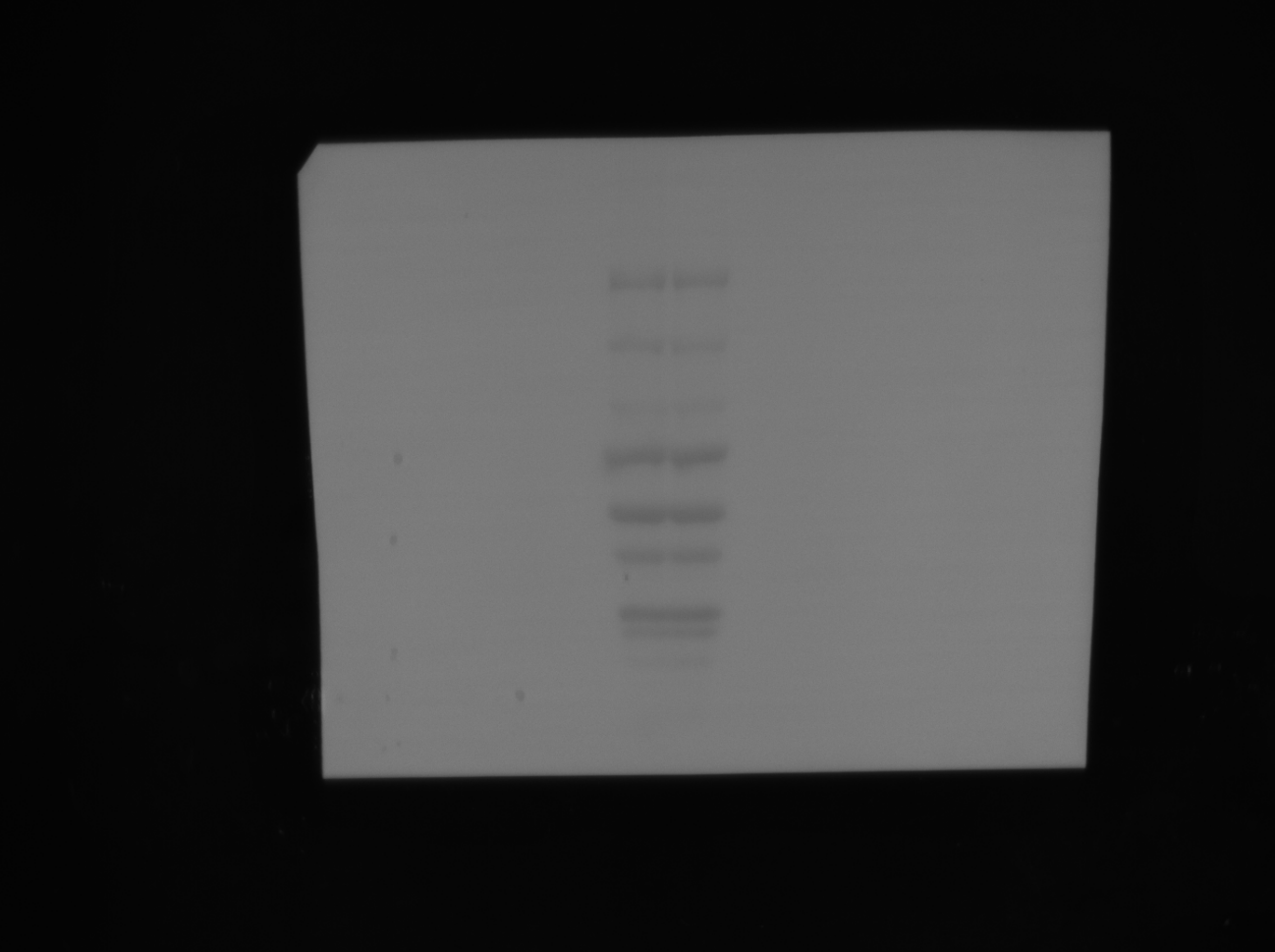


Figure S3.The original blot and gel of figure 2D.
